# Community awareness of stroke in Accra, Ghana

**DOI:** 10.1186/1471-2458-14-196

**Published:** 2014-02-21

**Authors:** Eric S Donkor, Mayowa O Owolabi, Patrick Bampoh, Thor Aspelund, Vilmundur Gudnason

**Affiliations:** 1 College of Health Sciences, University of Ghana, Accra, Ghana; 2 Department of Medicine, University of Ibadan and University College Hospital, Ibadan, Nigeria; 3 Tamale Central Hospital, Tamale, Ghana; 4 Icelandic Heart Association Research Institute, Kopavogur, Iceland; 5 Centre for Public Health Sciences, University of Iceland, Reykjavik, Iceland

**Keywords:** Stroke, Risk factors, Warning signs, Brain, Accra

## Abstract

**Background:**

Community awareness of stroke, especially the risk factors and warning signs is important in the control of the disease. In sub-Saharan Africa, little is known about community awareness of stroke though the brunt of stroke is currently borne in this region. The aim of the study was to evaluate stroke awareness in Accra (capital city of Ghana) particularly, the risk factors and warning signs.

**Methods:**

This was a cross-sectional study involving systematic sampling of 63 households in each of the 11 sub metropolitan areas of Accra. A structured questionnaire was used to collect stroke awareness data from respondents randomly sampled in the selected households. Logistic regression analyses were done to identify predictors of the main outcome variables including recognition of stroke risk factors, stroke warning signs and the organ affected by stroke.

**Results:**

Only 40% (n = 277) of the 693 respondents correctly identified the brain as the organ affected in stroke. Similarly, less than half of the respondents could recognize any of the established stroke risk factors as well as any of the established stroke warning signs. Over 70% (n > 485) of the respondents either believed that stroke is a preventable disease, or lifestyle alterations can be made to reduce the risk of stroke, or stroke requires emergency treatment. In multivariate analysis, predictors of stroke awareness were: age <50 years (OR = 0.56, CI = 0.35-0.92, p = 0.021), presence of a stroke risk factor (OR = 2.37, CI = 1.52-3.71, p < 0.001) and Christian Religion (OR = 14.86, CI = 1.37-161.01, p = 0.03).

**Conclusion:**

Though stroke is perceived as a serious and preventable disease in Accra, community awareness of the risk factors and warning signs is sub-optimal. This indicates that community-based education programs to increase public awareness of stroke could contribute to decreasing the risk of stroke and to increasing the speed of hospital presentation after stroke onset.

## Background

Stroke is ranked as the second leading cause of death worldwide with an annual mortality rate of 5.5 million [1]. The burden of stroke does not only lie in the high mortality but the high morbidity also results in up to 50% of survivors being chronically disabled [1,2]. Thus stroke is a disease of immense public health importance with economic and social consequences. Until recently, stroke was a disease of the developed world. However, through the application of evidence based control measures, the burden of stroke has reduced drastically in many developed countries. In most Western European countries, death from stroke has declined by 30-50% since 1975 and this is most noticeable in countries like Iceland, Italy, Austria and Germany [1,2]. The burden of stroke seems to be shifting to the developing world and currently two-thirds of stroke mortality cases occur in Sub-Saharan Africa [1,3], where poverty, malnutrition and communicable diseases such as HIV/AIDS also exert their greatest toll [2].

Stroke is the most preventable neurological disease, and this is mainly because, many of its risk factors such as hypertension, high cholesterol, diabetes and smoking can be prevented either through healthier lifestyle choices or by medication [4]. In the management of stroke, speedy access to medical service is crucial, as delays result in poor outcome [5,6]. Studies have shown that lack of recognition of stroke warning signs is an important causal factor of delay in hospital reporting of stroke [7,8]. Thus increased community knowledge of stroke risk factors and warning signs is important in the control of the disease, and health educational programmes have been very useful in this regard. For instance, provision of an information booklet on stroke significantly increased the knowledge of risk factors and warning signs in a sample of hospitalised stroke patients and their carers [9]. Given that the brunt of stroke is currently borne in sub-Saharan Africa, it is particularly important to understand the trend of community knowledge of stroke in the region and the associated factors. Nevertheless, studies that have investigated community knowledge of stroke were carried out mainly in the western countries [10].

In Ghana there is an increasing burden of stroke, and the disease ranks among the top three most important causes of mortality [11,12]. Community surveys carried out in some parts of Ghana reported high prevalence of some stroke risk factors particularly hypertension (19-48%) [13,14], which may partly explain the trend of increasing stroke cases in the country. Additionally, the high burden of stroke in Ghana has been attributed to poor community awareness of the disease, though there is limited information on community awareness of stroke in the country. For example, there is no study on awareness of stroke warning signs in Ghana, and information on awareness of stroke risk factors appears to be limited to only hypertension [15]. Though studies have investigated community awareness of stroke in some sub-Saharan Africa, it is difficult to extrapolate the findings to other sub-Saharan African countries due to significant disparities in the findings [16-20]. In countries with high stroke mortality like Ghana, there is an urgent need to assess community awareness of the disease, as this would provide the basis and direction for the relevant health education on the subject. The aim of this study was to evaluate stroke awareness in Accra, including the risk factors, warning signs, and other basic features of the disease such as the organ affected by stroke.

## Methods

### The study area

The study was carried out in Accra, the capital city of Ghana, from August to November, 2012. Ghana is a lower middle income country with literacy rate of 71.5%, and life expectancy of 65 years [21-23]. Accra is located in South-Eastern Ghana and has a population of about two million people [21]. The city is highly urbanized and cosmopolitan. Politically, Accra is divided into 11 sub metropolitan areas, each of which has 5–8 suburbs. There are about 27 hospitals in Accra and a national health insurance scheme has been in operation since 2004. There is also a proliferation of herbal centres in Accra, which offer non-orthodox medical services for a wide range of diseases including stroke. The Ghana Heart Foundation, a non-governmental organization, is the main organization involved in health education of stroke and other cardiovascular diseases in Accra and other parts of Ghana.

### Sampling and data collection

This was a cross-sectional study with multi-stage sampling technique. First, one suburb was randomly selected in each sub-metropolitan area of Accra. Based on 95% confidence limits with an allowable error of 10%, 63 households were sampled in each selected surburb using a systematic sampling methodology. In each chosen household one person between 18 and 60 years of age was randomly selected for interview using a structured questionnaire (see Additional file 1). This age range was chosen to increase the likelihood of obtaining accurate responses from the study participants.

The study questionnaire was developed through a literature review of stroke awareness and hospital presentation in different geographical settings. It was pretested with twenty people to ascertain its validity. The final questionnaire comprised 4 sections and 21 items, most of which had been used in previous studies [8,18,24,25].

The first section investigated respondents’ knowledge on stroke, including recognition of the organ involved in the disease, risk factors, warning signs, information resources, awareness of any community stroke organization and stroke beliefs/perceptions. For the organ affected by stroke, respondents were asked to choose between the brain and heart, or mention any organ they thought was the correct answer. For recognition of stroke risk factors, respondents were presented with a list of factors and asked to identify those associated with stroke. The list included hypertension, high cholesterol, poor eating, heart disease, smoking, obesity, family history of stroke, diabetes, stress, lack of exercise and alcohol use. For recognition of stroke warning signs, respondents were presented with a list of clinical signs and asked to identify stroke warning signs. The list included numbness on one side, numbness on any side, weakness on one side, weakness on any side, shortness of breath, headache, vision problems, dizziness, slurred speech and unspecified pain. For information resources, the respondents were to indicate whether they had learnt about stroke or not. Those who responded in the affirmative were asked to further indicate their sources of stroke knowledge from a list including the internet, newspaper/magazine, school, medical book, radio station, television and healthcare professional. Concerning awareness of community stroke organization, the respondents were asked to indicate if they had ever come across a stroke campaign in Accra. Regarding stroke perceptions and beliefs, respondents were presented with six statements about stroke and asked to indicate whether in each case they agreed with the statement or not, or they were not sure. The statements included: 1. Stroke is a preventable disease, 2. Lifestyle alterations can be made to reduce the risk of stroke, 3. Stroke affects only the elderly, 4. Stroke is a top killer diseases in Accra, 5. Stroke requires emergency treatment. 6. Stroke is a spiritual illness caused by evil spirits or witches.

The second section of the questionnaire was about the respondents’ response to a stroke attack, and they were to indicate whether they would visit the hospital, pharmacy, herbalist, or wait for stroke symptoms to subside. The respondents were also given the option of stating a planned response which was not indicated in any of the above choices. The third section of the questionnaire was about respondents’ demographic details including age, gender, marital status, religion, education and income. The fourth section of the questionnaire was about respondents’ presence of stroke risk factors. Here the respondents were to indicate which of the following risk factors they had: hypertension, heart disease, previous stroke, diabetes, high cholesterol, smoking and alcohol.

The questionnaire was self-administered in English, and on the average it took 15 minutes to complete. For respondents who could not communicate in English, the questionnaires were administered by two research assistants who had been trained in the interview process and were fluent in various Ghanaian dialects as well as English. The interviewers provided no clues to questions except to offer clarifications where necessary. The study was approved by the Ghana Health Service Ethics Review Committee (ID NO. GHS-ERC: 03/07/11) and informed consent was obtained from the study participants.

### Data analysis

The study data was analyzed with the Statistical Package for the Social Science version 11 (SPSS Inc). First, descriptive analyses including frequencies and corresponding percentages were carried out on the study variables. Logistic regression analyses were done to identify predictors of the main outcome variables including recognition of stroke risk factors, stroke warning signs and the organ affected by stroke. For each regression model, response options for the dependent variable were categorized as either “know” or “do not know.” In the case of recognition of stroke risk factors/warning signs “know” corresponded to recognition of ≥1 established stroke risk factors/warning signs. The independent variables used in the regression analyses were gender, age, religion, income, educational level, and the occurrence of at least one stroke risk factor. Missing data were excluded in the logistic regression analysis. The significance of predictor variables were assessed by p values, odds ratios and confidence intervals from Wald statistics; p values *≤* 0.05 were considered significant.

## Results

A total of 693 inhabitants of Accra were sampled in the study and their demographic features, as well as cardiovascular risk factor profile are summarized in Table 1. The mean age of the study respondents was 36.8 ± 14.0 years, and majority of them were males (n = 374, 54%) and Christians (n = 554, 80%). Marital status of the respondents showed similar proportions for those married (n = 267, 38%) and those who were single (n = 28, 41%). Secondary level education was the commonest educational level attained by the respondents (n = 360, 52%) and the rate of illiteracy was 14% (n = 97). Twenty eight percent of the respondents (n = 194) were unemployed, and the most common income range was GHC 100–999 (USD 50–500) which corresponds to 39% (n = 270) of the respondents. Forty percent of the respondents (n = 274) had at least one stroke risk factor, and the commonest risk factor was alcohol intake (n = 118, 17%), followed by hypertension (n = 76, 11%).

**Table 1:** Demographic features and stroke risk factor profiles of the study participants

**Variable**	**Frequency**	**%**
***Age (mean = 36.8 ± 14.0 yrs)***		
< 50 years	554	80
≥ 50 years	139	20
***Gender***		
Male	374	54
Female	319	46
***Religion***		
Christian	554	80
Moslem	111	16
Traditional religion	21	3
Other	7	1
***Marital status***		
Married	267	38
Single	281	41
Widowed	68	10
Separated	50	7
Divorced	27	4
***Highest education attained***		
Illiterate	97	14
Primary	104	15
Secondary	360	52
Tertiary	132	19
***Monthly income (Cedis)***		
< 100	180	26
100-999	270	39
1000-1999	21	3
2000-2999	14	2
≥ 3000	14	2
Unemployed	194	28
***Stroke risk factors***		
Alcohol intake	118	17
Hypertension	76	11
Smoking	47	7
Previous stroke	43	6
High cholesterol	25	4
Heart disease	19	3
Diabetes	17	2
At least one stroke risk factor	274	40

Respondents’ recognition of stroke risk factors and warning signs are reported in Table 2. Lack of exercise was the commonest factor cited by the respondents as a stroke risk factor (n = 256, 37%), while diabetes was least cited as a stroke risk factor (n = 90, 13%). In the case of stroke warning signs, numbness on one side was the commonest warning sign cited by the respondents (n = 304, 44%) while unspecified pain was the least cited (n = 79, 11%). Analysis of correct answers provided by the respondents showed that, 159 (23%) correctly listed one established stroke risk factor, 145 (21%) correctly listed two established risk factors, 97 (14%) correctly listed three established risk factors, and 69 (10%) correctly listed four or more established risk factors for stroke. In the case of warning signs, 132 (19%) respondents correctly listed one established stroke warning sign, 139 (20%) correctly listed two established warning signs, 111 (16%) correctly listed three established warning signs, and 159 (23%) correctly listed four or more established warning signs for stroke. In the multiple logistic regression analysis (Table 3), having a stroke risk factor (OR = 2.37, CI = 1.52-3.71, p < 0.001) and Christian Religion (OR = 14.86, CI = 1.37-161.01, p = 0.03) were associated with higher levels of awareness of stroke risk factors. However, no significant associations were observed between awareness of stroke warning signs and any of the explanatory variables investigated.

**Table 2:** Knowledge of stroke risk factors and warning signs of the study participants

**Variable**	**Frequency**	**%**
***Risk factors***		
*Lack of exercise	256	37
*Hypertension	239	34
*Alcohol	231	33
*High Cholesterol	222	32
Family history of stroke	194	28
*Smoking	167	24
Stress	155	22
*Heart disease	125	18
Obesity	97	14
Poor eating	83	12
*Diabetes	90	13
No knowledge of risk factors	139	19
***Warning signs***		
*Numbness (on one side)	304	44
*Weakness (on one side)	266	38
*Slurred speech	256	37
*Severe headache	173	25
*Numbness (on any side)	147	21
*Dizziness	117	17
*Weakness (on any side)	104	15
*Vision problems	103	15
Shortness of breath	90	13
Unspecified pain	79	11
No knowledge of warning signs	152	22

*established stroke risk factor or warning sign.

**Table 3:** Predictors of stroke awareness through multivariate logistic regression

**Variable**	**Risk factors**		**Warning signs**		**Affected organ**	
	**p**	**OR (95% CI)**	**p**	**OR (95% CI)**	**p**	**OR (95% CI)**
Age <50 years	0.87	0.96 (0.57-1.61)	0.92	1.03 (0.59-1.80)	0.02	0.57 (0.35-0.92)
Male gender	0.21	1.28 (0.87-1.88)	0.95	0.99 (0.65-1.49)	0.37	1.18 (0.82-1.72)
Christian religion	0.03	14.86 (1.37-161.01)	1.00	0.00 (0.00-23.09)	0.51	2.20 (0.21-23.30)
Tertiary level of education	0.26	1.58 (0.71-3.52)	0.25	1.66 (0.70-3.92)	0.09	1.89 (0.91-3.91)
Monthly income > GHC 3,000	0.16	4.8 (0.54-43.05)	0.37	2.67 (0.31-22.84)	0.08	4.33 (0.84-22.33)
Presence of a stroke risk factor	<0.01	2.37 (1.52-3.71)	0.06	1.57 (0.98-2.52)	0.10	1.41 (0.94-2.10)

When asked which organ of the body was associated with stroke, 40% (n = 277) of the respondents correctly mentioned the brain, 10% (n = 69) mentioned the heart, 44% (n = 305) mentioned other parts of the body which are not organs at all, and 6% (n = 42) indicated they did not know the answer. In the multiple logistic regression analysis (Table 3), identification of brain as the organ affected by stroke was significantly associated with younger age, that is age <50 years (OR = 0.56, CI = 0.35-0.92, p = 0.021).

Responses of the study participants to the six stroke beliefs and/or perceptions investigated are summarized in Table 4. Over 70% (n > 485) of the respondents either believed that lifestyle alterations can be made to reduce the risk of stroke, or stroke is a preventable disease, or stroke requires emergency treatment. Forty five percent of the respondents (n = 312) agreed that stroke is one of the top killer diseases in Accra. The beliefs that stroke affects only the elderly and stroke is a spiritual illness caused by evil spirits or witches were shared by 26% (n = 180) and 25% (n = 173) of the respondents respectively.

**Table 4:** Stroke beliefs/perceptions investigated among the study participants

**Stroke belief/perception**	**Yes**		**No**		**Not sure**	
	**n**	**%**	**n**	**%**	**n**	**%**
Lifestyle alterations can be made to reduce the risk of stroke	575	83	35	5	83	12
Stroke is a preventable disease	554	80	35	5	104	15
Stroke requires emergency treatment	536	77	46	7	113	16
Stroke is a top killer diseases in Accra	312	45	118	17	263	38
Stroke affects only the elderly	180	26	374	54	139	20
Stroke is a spiritual illness caused by evil spirits or witches	173	25	333	48	187	27

In the event of a stroke, 520 (75%), 55 (8%) and 14 (2%) of the respondents planned to visit the hospital, herbalist and pharmacy respectively. Sources of stroke information reported by the respondents were as follows: radio station- 277 (40%), television- 222 (32%), healthcare professional- 159 (23%), school- 69 (10%), medical textbook- 69 (10%), internet- 62 (9%), newspaper/magazine- 62 (9%), family/friends- 28 (4%), other sources- 35 (5%); a total of 180 (26%) of the respondents indicated that they had never learned about stroke. When asked whether the respondents had ever come across a stroke campaign in Ghana, 86% (n = 596) responded in the negative, while 14% (n = 97) responded in the affirmative.

## Discussion

Data from this study indicates that generally, stroke is perceived as a serious illness (requires emergency treatment), is preventable and lifestyle alterations can be made to reduce its risk. However, the data also shows that there is poor community awareness of stroke risk factors and its warning signs. Poor awareness of stroke risk factors in this study is shown by the fact that, hypertension which is the most important stroke risk factor in Ghana [26,27], could not be recognized by majority (66%) of the respondents. This is also the case of major risk factors of stroke such as high cholesterol, diabetes, alcohol and smoking. “Lack of exercise” was the commonest risk factor cited by the respondents, and is probably because exercising is one of the major stroke rehabilitation methods, and thus it is commonly perceived that people who do not exercise are at risk of stroke. Poor community awareness of stroke risk factors has also been reported in Benin [16] and Nigeria [20]. However in Nigeria, a study among university staff and students reported appreciable level of awareness of stroke risk factors with majority of the respondents correctly identifying each of the major stroke risk factors [28]. Similar studies done outside Africa, have also reported poor community knowledge of stroke risk factors, and this is evident in data from Brazil [8], Ireland [24], Australia [25], Pakistan [29] and the United States [30]. Studies have shown that increased awareness of stroke risk factors among people at high risk for stroke leads to improved compliance with stroke prevention practices [31,32]. In Ghana there is currently a stroke epidemic [11], and this could partly reflect the poor community awareness of stroke risk factors observed in this study.

Majority of the respondents could not recognize any of the basic stroke warning signs such as slurred speech and vision problems, an observation which has also been reported by studies carried out in both developed and developing countries [8,16,18,24,25]. In a stroke burdened country like Ghana, poor community knowledge of stroke warning signs is of serious concern, as recognition of the warning signs is a predictor of early hospital reporting of the disease [5,6]. Although a large proportion of the respondents (75%) indicated that they would visit the hospital in the event of a stroke attack, this could be affected unfavourably by the poor recognition of stroke warning signs observed. Numbness or paralysis was the commonest stroke warning sign in this study, which concurs with studies done in Nigeria [18] and Benin [16] but contrasts with studies in Australia [25] and Ireland [24] where vision problems and slurred speech respectively were the commonest warning signs identified.

In addition to poor community awareness of stroke risk factors and warning signs in Accra, there are misconceptions about the organ involved in stroke, with only 40% of the respondents correctly identifying the brain as the organ affected by stroke; by comparison similar studies reported 14% in Benin [16], 73% in Australia [25] and 52% in Pakistan [28]. In terms of the numbers of correct responses, we observed similar levels of knowledge of stroke risk factors and warning signs, unlike most studies which showed that respondents’ knowledge of warning signs were significantly poorer than risk factors [24,25,30]. Predictors of stroke knowledge in this study also appear to be different from those of some other studies. While income and education were determinants of knowledge of stroke risk factors in studies carried out in Australia [25], Brazil [8] and Ireland [24], this was not the case with the Ghanaian data. Having a stroke risk factor was a strong determinant in the recognition of stroke risk factors in this study, and has also been reported in Ireland [24] but not Brazil [8]. These observations highlight the geographical and cultural variations in determinants of stroke knowledge, and may be important in planning health education of stroke. Though Christian religion was identified to be associated with increased awareness of stroke risk factors (OR 14.86), the large confidence interval of 1.37-161.01 will make one to be careful in interpreting this odd ratio even though it is statistically significant (p = 0.03).

This study has not only revealed the poor community awareness of stroke risk factors in Accra, but has also shown a high general prevalence of self-reported risk factors (40%) which may explain the high morbidity of stroke in the city. Though stroke is one of the three most common causes of mortality in Ghana currently, minority of the respondents (45%) were aware of the fact the disease is a top killer. This is probably due to poor publicity given to the disease in Ghana compared to other diseases such as Malaria. Evidence partly in support of this is the fact that >80% of the respondents had never come across a stroke campaign in Ghana. Generally, the findings of the study highlight the necessity of public health education of stroke in Accra, and probably the whole of Ghana. Such a programme should aim primarily at improving community awareness of stroke risk factors and warning signs, and also encouraging behavioural changes that would decrease risk of the disease. In this study, over 70% of the respondents had learnt some information about stroke through the radio or television, indicating that the two media would be very useful for dissemination of stroke information in a public health education programme.

## Conclusions

We conclude that, though stroke is perceived as a serious and preventable disease in Accra, community awareness of the risk factors and warning signs is sub-optimal. We hypothesize that this reflects the trend in the whole of Ghana and is partly responsible for the high morbidity and mortality of stroke in the country. Community-based education programs to increase public awareness of stroke could contribute to decreasing the risk of stroke and to increasing the speed of hospital presentation after stroke onset.

## Competing interests

The authors declare that they have no competing interests.

## Authors’ contributions

ESD, MOO, PB, TA and VG conceived and designed the study. ESD, PB and MOO organized data collection. ESD, TA and VG undertook data analysis. ESD drafted the manuscript and all authors contributed substantially to its revision. All authors read and approved the final manuscript.

## Pre-publication history

The pre-publication history for this paper can be accessed here:

http://www.biomedcentral.com/1471-2458/14/196/prepub

## Supplementary Material

Additional file 1 Study questionnaire: community awareness of stroke in Accra. Click here for file
