# Supplementary material for: Community awareness of stroke in Accra, Ghana
Source: BMC Public Health. 2014 Feb 21;14:196. doi: 10.1186/1471-2458-14-196 (PMC3943505; doi:10.1186/1471-2458-14-196)
Supplement: Additional file 1 — Study questionnaire: community awareness of stroke in Accra. [file 1471-2458-14-196-S1.doc]

**Additional file 1**

**STUDY QUESTIONNAIRE: COMMUNITY AWARENESS OF STROKE IN ACCRA**

**Date: _____________ Area _____________________**

**Respondent’s ID**_______________________________

**Sex**: □ Male □ Female

**Age**_____________________

**Highest education completed**

□ Primary □ Secondary □ Tertiary □ None

**Marital status**

□ Married □ Single □ Separated

□ Divorced □ Widowed

**Religion**

□ Christian □ Moslem □ Traditional religion

□ Other (specify) ________________

**Monthly income in Ghanaian Cedis**

□ < 100 □ 100-999 □ 1,000-1,999

□ 2,000-2,999 □ ≥3,000 □ Not working

**1. Which of the following apply to you?**

□ Hypertension □ Cholesterol

□ Previous Stroke □ Smoking

□ Diabetes □ Drinking more than 2 glasses of alcohol a day

□ Heart disease □ None of the listed conditions

**2. Stroke affects which organ of the body?**

□ Brain □ Heart □ Other (specify) ________________________

**3. Which of the following do you think are potential risk factors of stroke? (you can choose more than one)**

□ Hypertension □ Smoking □ Stress

□ Cholesterol □ Obesity □ Lack of exercise

□ Poor eating □ Family history of stroke □ Alcohol use

□ Heart disease □ Diabetes □ Do not know

**4. What are the warning symptoms of stroke? (you can choose more than one)**

□ Numbness (1 side) □ Shortness of breath □ Slurred speech

□ Numbness (any) □ Headache □ Pain, unspecified

□ Weakness (1 side) □ Vision problems □ Do not know

□ Weakness (any) □ Dizziness

**5.** **What would be your cause of action in the event of a stroke?**

□ Visit the hospital □ Visit the pharmacy □ Visit the herbalist

□ Wait and observe symptoms to see if they subside

□ other____________________

**6. Do you believe that............**

Stroke is a preventable disease

□ Yes □ Not sure □ No

Lifestyle alteration can be made to reduce the risk of stroke

□ Yes □ Not sure □ No

Stroke affects only the elderly

□ Yes □ Not sure □ No

Stroke is one of top killer diseases in Accra

□ Yes □ Not sure □ No

Stroke requires emergency treatment

□ Yes □ Not sure □ No

Stroke is a spiritual illness caused by evil spirits or witches

□ Yes □ Not sure □ No

**7. Which of the following sources have provided you with your knowledge of stroke? (please tick all that apply)**

□ Never learned about stroke □ Medical books

□ Internet □ Radio station

□ Newspaper/magazine □ Television

□ School □ Healthcare professionals

□ Other (please state)____________________________________

**8. Have you ever come across a stroke campaign?**

□ Yes

□ No
